# *Spodoptera frugiperda* Salivary Glucose Oxidase Reduces the Release of Green Leaf Volatiles and Increases Terpene Emission from Maize

**DOI:** 10.3390/insects15070511

**Published:** 2024-07-08

**Authors:** Bin Gao, Bin Li, Jinxi Yuan, Zhan Shi, Xialin Zheng, Guirong Wang

**Affiliations:** 1 Guangxi Key Laboratory of Agri-Environmental and Agri-Products Safety, College of Agriculture, Guangxi University, Nanning 530004, China; gaob1n@163.com; 2 Shenzhen Branch, Guangdong Laboratory of Lingnan Modern Agriculture, Genome Analysis Laboratory of the Ministry of Agriculture and Rural Affairs, Agricultural Genomics Institute at Shenzhen, Chinese Academy of Agricultural Sciences, Shenzhen 518120, China; 82101209119@caas.cn (B.L.); yuanjinxi1022@163.com (J.Y.); shizhan0522@163.com (Z.S.); 3 Key Laboratory of Pest Monitoring and Green Management, Ministry of Agriculture and Rural Affairs, Department of Entomology, China Agricultural University, Beijing 100091, China; 4 School of Life Sciences, Henan University, Kaifeng 475004, China

**Keywords:** *Spodoptera frugiperda*, glucose oxidase, HIPVs, GC-MS, green leaf volatiles

## Abstract

**Simple Summary:**

*Spodoptera frugiperda* is a significant global agricultural pest, particularly fond of maize. In agroecosystems, the interactions among plants, phytophagous insects, and their natural enemies form a complex web at three trophic levels, with volatile compounds playing a pivotal role in these interactions. Phytophagous insects utilize plant volatiles as chemical cues to accurately identify and locate their hosts. Furthermore, herbivore-induced plant volatiles (HIPVs) act as critical chemical messengers and play an essential role in mediating interactions across the three trophic levels. These interactions include inducing defense mechanisms in neighboring plants, attracting natural enemies to help suppress pest populations, or directly producing repellent substances to deter pests. Oral secretions (OS) play a vital role in this interaction, acting as a critical signaling conduit that modulates plant responses. In this study, the major agricultural pest *S. frugiperda* was examined, with the *GOX* gene targeted as a molecular focus. The findings indicate that the *GOX* gene influences HIPV emission in maize, providing valuable insights into plant–insect interaction mechanisms and laying a foundation for innovative plant protection strategies.

**Abstract:**

The intricate relationships between plants and insects are essential for understanding ecological dynamics. Among these interactions, HIPVs serve as a pivotal defense mechanism. Our findings reveal the highly conserved nature of the *GOX* gene within the Lepidoptera order, highly expressed in the salivary glands of *S. frugiperda*, and its role in mediating maize’s defense responses. Notably, salivary GOX activity expression significantly decreases subsequent gene knockout. The presence of GOX in the saliva of *S. frugiperda* significantly modulates the emission of HIPVs during maize consumption. This research delineates that GOX selectively inhibits the emission of certain green leaf volatiles (GLVs) while concurrently enhancing the release of terpene volatiles. This study unveils a novel mechanism whereby *S. frugiperda* utilizes GOX proteins in OS to modulate volatile emissions from maize, offering fresh perspectives on the adaptive evolution of phytophagous insects and their interactions with their preferred host plants.

## 1. Introduction

*Spodoptera frugiperda* (fall armyworm) is an important Lepidopteran pest. This omnivorous insect can feed on more than 350 host plant species, including important staple crops such as maize (*Zea mays*), rice (*Oryza sativa*), and sorghum (*Sorghum bicolor*) [1,2]. Indigenous to tropical and subtropical regions of the Americas, this pest invaded Africa in 2016 and spread across the globe since then, posing a severe threat to local and global food security [3,4,5]. Current management regimes of *S. frugiperda* primarily rely on the adoption of transgenic crops expressing *Bt* toxins, while in regions where this technology is not accessible, heavy application of chemical pesticides becomes the predominant pest control measure [6]. The efficiency and sustainability of the current management strategies for *S. frugiperda* can be enhanced by the development and adoption of pest-resistant crop cultivars.

Maize is the favored host plant of *S. frugiperda* [7]. Mechanistic investigation of how maize plants naturally defend themselves against *S. frugiperda* and how this maize-specialized insect herbivore adapts towards these defense mechanisms can facilitate the genetic enhancement of maize resistance against *S. frugiperda*. For example, it has been reported that *S. frugiperda* can tolerate benzoxazinoids, the major defensive metabolites of maize, by re-glycosylation [8]. Emission of herbivore-inducible plant volatiles (HIPVs), including fatty acid-derived green leaf volatiles (GLV) and simple terpenoids, is an important component of plant defense [9]. These HIPVs can serve as long-distance airborne signals to attract natural enemies of insect herbivores and warning cues to prime defense responses in neighboring plants, or even be directly toxic against the herbivores themselves [10]. In maize, *S. frugiperda* attacks have been shown to elicit a significantly lower level of HIPV release compared to other chewing Lepidopteran pests, which has contributed to the success of this insect in maize [11].

The lower inducible defense response of maize towards *S. frugiperda* has been associated with various constituents of the oral secretion (OS) of this insect herbivore. The insect OS is a mixture of secretions from salivary and accessory glands, along with gut regurgitant [12]. Among Lepidopteran herbivores, diverse small molecules and larger peptides or proteins have been shown to either elicit or suppress plant defense [13,14]. For *S. frugiperda* specifically, the salicylic acid in its OS has been proposed to suppress plant defense by antagonizing the jasmonic acid signaling [15]. A phospholipase C in *S. frugiperda* OS can induce defense responses in maize and Bermuda grass and reduce caterpillar weight gain [16]. Additionally, bacteria found in the OS could also have plant defense-suppressing functions [17].

In many Lepidopteran herbivores, the glucose oxidase (GOX) enzymes in their OS have been reported to play important roles in affecting plant inducible defense. For example, the GOX in *Helicoverpa zea* OS can inhibit the production of nicotine in tobacco, as well as induce stomata closure and reduce the release of HIPVs in tomato [18]. The GOX of *Ostrinia nubilalis* can elicit JA pathway and late-responding defenses in tomato [19,20]. Together, these studies suggest that the function of GOX in plant–insect herbivore interaction can be highly variable in different plant and insect species.

In this study, we investigated the function of the GOX found in the OS of *S. frugiperda* larvae in maize—*S. frugiperda* interaction. Genetic knock-out of *SfruGOX* did not impact larvae growth when using either artificial diet or maize seedlings. However, the expression of genes involved in GLV biosynthesis and the GLV contents were significantly elevated when maize plants were attacked by the *SfruGOX*^−/−^ mutant larvae compared to wild-type larvae. In contrast, terpene volatile contents and their related biosynthetic gene expression were significantly suppressed upon *SfruGOX*^−/−^ mutant larvae feeding. These findings reveal the complex function of *SfruGOX* in regulating host plant defense response and may provide a novel perspective on *S. frugiperda* management.

## 2. Materials and Methods

### 2.1. Insect and Plant Materials

The fall armyworm was kindly supplied by Dr. Yutao Xiao at the Agricultural Genomics Institute at Shenzhen (AGIS). They were raised on artificial feed in an insect breeding room, with an ambient temperature of 26 ± 1 °C, relative humidity of 60–70%, and light conditions of 14L:10D (dark cycle starting at 20:00). Seeds of the inbred maize line B73 were originally obtained from Dr. Shaoqun Zhou at the AGIS, and were maintained as previously described [21].

### 2.2. Sequence Alignment and Phylogenetic Analysis

The functionally characterized GOX protein of *Spodoptera exigua* was the query to blast in the genome of *S. frugiperda* [22]. The sequences with similarities of over 75% were collected to build phylogeny trees in order to determine the orthologous relationship. The identified SfruGOX amino acid sequence was aligned with selected GOX sequences of *H. zea*, *Spodoptera litura*, *S*. *exigua*, *H. armigera*, *Heliothis viriplaca*, *Chilo suppressalis*, *Ostrinia furnacalis*, *Mythimna separata,* and *Bombyx mori* using DNAMAN 8.0 software. Homologous GOX sequences from various insects were retrieved from GenBank, and their accession numbers are detailed in Table S1. A phylogenetic tree of the GOXs was constructed using MEGA 7.0 software, employing the neighbor-joining method.

### 2.3. RNA Extraction, cDNA Synthesis, and qRT-PCR

The total RNA of FAW larvae tissues and maize leaves was extracted with the TRIzol protocol and reverse-transcribed using the HiScript^®^ III 1st Strand cDNA Synthesis Kit (+gDNA wiper) (Vazyme, Nanjing, China). The qPCR was performed on a CFX-96™ PCR Detection System (Bio-Rad, Hercules, CA, USA) with the reagent of Taq Pro Universal SYBR qPCR Master Mix (Vazyme, Nanjing, China) employed within a 10 µL reaction volume. This reaction mixture comprised 5 µL of 2× ChamQ Universal SYBR^®^ qPCR Master Mix, 0.5 µL of both forward and reverse primers (10 µM), 1 µL of the cDNA template, and 3 µL of RNase-free water. The thermal cycling conditions were set as follows: initial denaturation at 95 °C for 3 min, followed by 39 cycles of 95 °C for 10 s and 55 °C for 30 s, a final extension step at 65 °C for 5 s, and a melt curve analysis starting from 65 °C to 95 °C. using 2^−ΔΔCT^ for data processing. The maize reference genes were EF1a and A-TUB, and the insect reference genes were RPL10, a-TUB, and SOD. Quantitative analysis of each instar and tissue of *S. frugiperda* larvae and of maize leaves fed on by *S. frugiperda* larvae, with 3–4 replicates in each group, was conducted. Primers for qPCR were designed using the NCBI website (https://www.ncbi.nlm.nih.gov/, accessed on 20 February 2023). All primers are listed in the Supplementary Materials (Table S2).

### 2.4. CRISPR/Cas9-Based Knock out of SfruGOX

To generate mutants of the *GOX* gene, we employed the CRISPR/Cas9 genome editing system. Specific primers were designed to amplify exon 2 of the *GOX* gene. The resulting PCR product was ligated into a BLUNT vector, followed by sequencing to confirm sequence integrity and conservation. To identify highly efficient sgRNA targets within conserved regions of the *GOX* gene, we utilized the CRISPOR online software (http://crispor.tefor.net/) for prediction analyses [23]. Based on these analyses, sgRNAs were synthesized utilizing the GeneArt gRNA Synthesis Kit (Thermo Fisher Scientific, Waltham, MA, USA). Sexually mature females and males were pre-selected and housed together in mating cages to maximize embryo production. To facilitate egg laying, adults were transferred to a dark environment, and moistened filter paper was positioned atop the oviposition container. Eggs were monitored at 10 min intervals, ensuring the prompt collection of freshly laid embryos. The collected embryos were meticulously aligned on microinjection plates and subjected to microinjection employing an Eppendorf InjectMan 4 system. The injection was completed within 1 h of oviposition, and about 400 eggs were injected. The injection solution comprised Cas9 protein at a concentration of 150 ng/μL and sgRNA at 300 ng/μL. G0 embryos underwent injection and were subsequently incubated under controlled conditions (26 ± 1 °C temperature and 60–70% relative humidity) within a dedicated worm-rearing chamber. Upon the emergence of G0 generation, mid-leg samples were collected for mutation analysis. G0 chimeras were mated individually with wild-type counterparts to produce G1 offspring. G1 individuals harboring identical mutations were interbred to establish a homogeneous line in the G2 generation. In cases where no consistent mutation type was identified in G1, heterozygous mutants were outcrossed to wild types, aiming to secure a pure line through G3 generation.

### 2.5. The Salivary GOX Activity Assay

To evaluate the impact of *GOX* knockout on GOX enzyme activity in the salivary glands of *S. frugiperda* larvae, we measured GOX activity using the Glucose Oxidase Activity Detection Kit (Beijing Solepole Co., Ltd., Beijing, China). Prior to conducting the experiments, fifth-instar larvae from both the wild-type and *SfruGOX*^−/−^ genotypes were subjected to a 6 h fasting period, followed by feeding on B73 corn leaves. Twelve hours post-feeding, salivary glands, weighing approximately 0.1 g, were excised, immediately frozen in liquid nitrogen, and stored until analysis. For each genotype, nine biological replicates were prepared, with each replicate comprising tissue from 10–15 larvae. Tissue samples were homogenized in 1 mL of extraction buffer provided in the kit, following a standardized protocol to ensure consistency. The homogenization process was performed in an ice bath to preserve enzyme integrity. Subsequently, the GOX enzyme activity was quantitatively assessed according to the manufacturer’s instructions provided in the Solebo Glucose Oxidase Activity Assay Kit (Beijing Solepole Co., Ltd., Beijing, China).

### 2.6. Insect Performance Bioassay

For the bioassay experiment, B73 maize seedlings aged 12–18 days and first-instar larvae were selected. In each nursery pot, two maize plants were positioned at opposite corners. A transparent PVC enclosure (dimensions: 5 cm × 5 cm × 30 cm) equipped with air circulation holes was placed over the second leaf of each corn plant. Two first-hatch larvae were introduced onto the leaf within each sealed enclosure. The experiment was conducted with approximately 62 larvae for each genotype (wild-type and *SfruGOX*^−/−^). After seven days of feeding, the larvae were weighed to assess growth. Concurrently, a control group was established using larvae reared on an artificial diet in 24-well plates, totaling 42 individuals per genotype. This setup aimed to compare the body weight differences between WT and *SfruGOX*^−/−^ larvae after consuming either maize leaves or an artificial diet.

### 2.7. Transcriptome Sequencing of Maize Leaves Attacked by S. frugiperda Larvae

Having been starved for 6 h, fifth-instar *S. frugiperda* larvae were individually placed on the second fully expanded leaves of 12–18-day B73 maize plants. After a feeding period of 6 h, samples were collected, with each replicate weighing between 100 and 150 mg. A total of four replicates per treatment were prepared. These samples were used for RNA-seq library preparation using Beijing Novogene Bioinformatics Technology (Beijing, China). The transcriptome data analyses were carried out as previously described [24]. Briefly, cleaned PE150 reads were mapped to maize B73 reference genome v4 using STAR 2.4.0j software, and differentially-expressed genes were identified with DEseq2 (1.20.0) [25,26,27]. Kyoto Encyclopedia of Genes and Genomes (KEGG) pathway enrichment analyses of the DEGs were performed with the R package GSEABase and GOstats [28,29,30].

### 2.8. GC-MS Detection of Volatile Substances

In this study, fifth-instar larvae that were starved for 6 h were selected to be placed on 14–18-day-old corn leaves for feeding, and the prepared corn seedlings were placed in sealable glass jars (diameter = 22 cm, height = 55 cm) for volatile collection. Samples were collected through dynamic headspace collection using superQ adsorbent from 21:00 to 09:00, and collection lasted for 12 h. The experimental method was carried out as described by previous authors [31,32,33]. Gas chromatography–mass spectrometry (GC-MS) analysis was conducted using a Ther-mo TSQ9000+Trace1310 gas chromatograph equipped with a DB-5MS UI column (30 m × 0.25 mm × 0.25 µm, Agilent, Santa Clara, CA, USA). The carrier gas was helium, set at a flow rate of 1.2 mL/min, with an inlet temperature of 250 °C and no split injection. The thermal program began at an initial temperature of 40 °C and was held for 5 min, followed by a ramp to 290 °C at a rate of 5 °C/min, where it was held for an additional 10 min. Mass spectrometry conditions included: electron ionization (EI) at 70 eV, helium as the carrier gas at a flow rate of 1.2 mL/min, transfer tube temperature at 250 °C, ion source temperature at 300 °C, and a scanning range of 50–500 *m*/*z*.

### 2.9. Statistical Analyzes

Data were presented as mean ± SE and analyzed using GraphPad Prism v9.4.1. Statistical analyses were conducted using Student’s *t*-test or one-way ANOVA. *p*-values lower than 0.05 were considered statistically significant.

## 3. Results

### 3.1. GOX Genes Are Conserved in Lepidoptera and Highly Expressed in the Salivary Glands of the S. frugiperda Larvae

**Figure 1:**
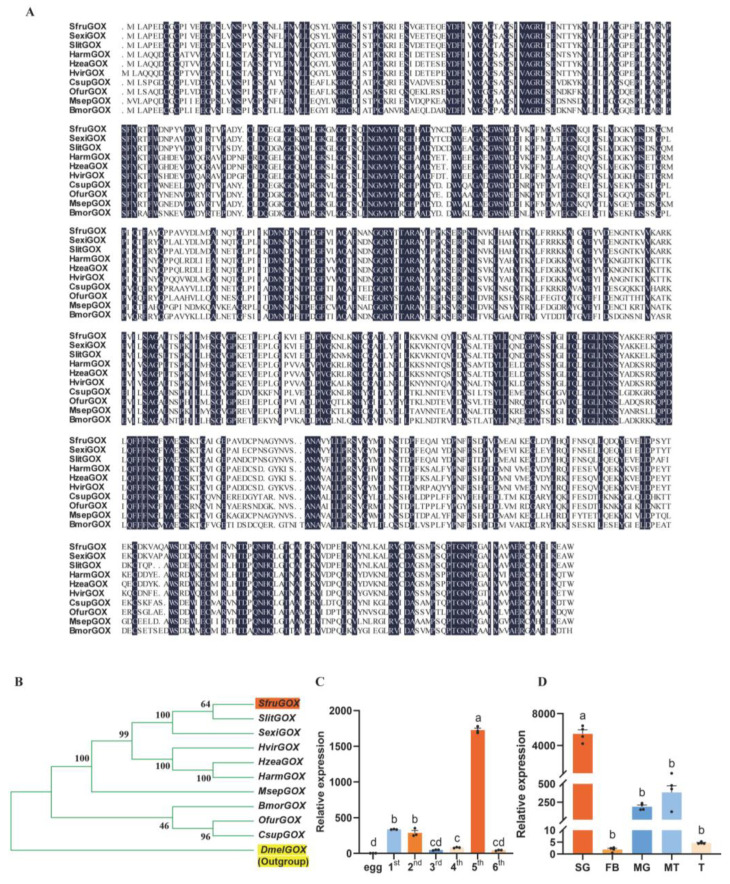
Sequence analysis and expression of *GOX* gene. (**A**) Amino acid sequences of *S. frugiperda* GOX were compared with those of *H. zea*, *S. litura*, *S. exigua*, *H. armigera*, *H. viriplaca*, *C. suppressalis*, *O. furnacalis*, *M. separata*, and *B. mori*. Conserved amino acids are highlighted in dark blue. (**B**) Phylogenetic analyses of *S. frugiperda*, *H. zea*, *S. litura*, *S. exigua*, *H. armigera*, *H. viriplaca*, *C. suppressalis*, *O. furnacalis*, *M. separata*, and *B. mori* using MEGA; Orange markers are *S. frugiperda* and yellow markers are the outgroup *D. melanogaster*. (**C**) *SfruGOX* gene expression in larvae of different instars (Note: egg: egg; 1st: 1st-instar larva; 2nd: 2nd-instar larva; 3rd: 3rd-instar larva; 4th: 4th-instar larva; 5th: 5th-instar larva; 6th: 6th-instar larva; mean ± SE from three replicates is shown. Different letters above bars indicate significant differences (*p* < 0.05) according to Student’s *t*-test). (**D**) *SfruGOX* gene expression analysis in different larval tissues (Note: SG: salivary gland; FB: fat body; MG: midgut; MT: Malpighian tubule; T: testes; mean ± SE from four replicates is shown. Different letters above bars indicate significant differences (*p* < 0.05) according to Student’s *t*-test).

In congruence to the homology-based annotation, the predicted peptide sequence of SfruGOX showed 93.26% identity with the functionally characterized GOX protein of *S. exigua* [22]. A homology search with the SfruGOX peptide sequence as the query in ten lepidopteran species and *Drosophila melanogaster* revealed that this gene was a conserved gene across these species, and the structure of the gene family tree was consistent with the phylogenetic relationship among these species (Figure 1A,B). It is implied that it may have similar functions.

The GOX has been detected in the saliva of various Lepidoptera insects, including *H. zea* [34]. To determine the presence and tissue-specific expression of the *GOX* gene in *S. frugiperda* and to explore the association between *GOX* gene expression and the feeding behavior of *S. frugiperda*, we performed qRT-PCR analyses on different developmental stages and tissues of *S. frugiperda*. The result showed that *GOX* gene expression was highest in the fifth-instar larvae of *S. frugiperda* (Figure 1C). Furthermore, tissue-specific qRT-PCR experiments showed that *SfruGOX* was highly expressed in the salivary glands of fifth-instar larvae (Figure 1D). These findings suggest that secretion of *GOX* through the mouthpart during feeding is likely a conserved physiological response across Lepidopteran herbivores.

### 3.2. The Absence of GOX Did Not Affect Larvare Growth

**Figure 2:**
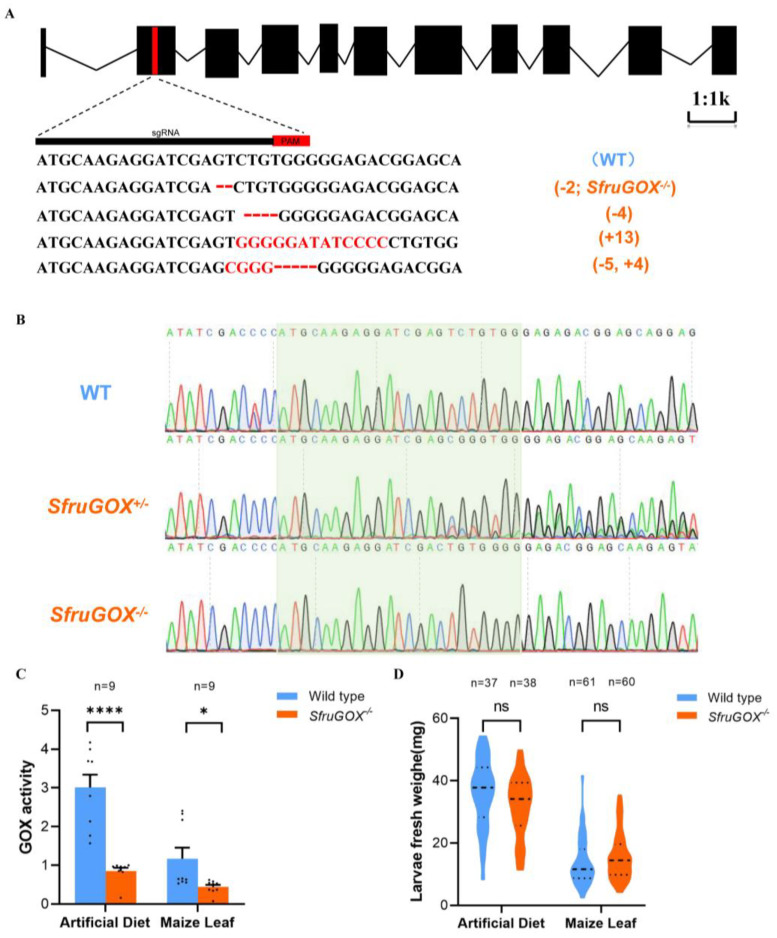
Genotypes and phenotypes of *S. frugiperda* mutants. (**A**) Sequences of insertion–deletion mutations in the larvae of G0 generation. The WT sequence is shown at the top, with the target site and the PAM is marked in red. In mutant sequences, deletions are shown as dashes and insertions as red letters. (**B**) Plot of sequencing analysis of PCR products of WT, *SfruGOX*^+/−^ heterozygote, and *SfruGOX*^−/−^ mutant. The heterozygous *SfruGOX*^−/−^ mutant sequence shows sets of peaks, indicating a mutation. *SfruGOX*^−/−^ is a pure mutant. (**C**) Salivary gland glucose oxidase (GOX) activity. (Maize leaf, *t* = 2.505, df = 16, *p* = 0.0235, artificial diet, *t* = 6.367, df = 16, *p* < 0.0001; *, *p* < 0.05, ****, *p* < 0.0001). (**D**) Comparison of body weights of wild-type and mutant *S. frugiperda* feeding on artificial fodder and B73 maize for 7 days. (Artificial diet, *t* = 1.838, df = 73, *p* = 0.0701, maize leaves *t* = 1.386, df = 119, *p* = 0.1682; ns, not significance).

Previous literature has shown that GOX proteins in OS play a critical role in Lepidopteran herbivores [18,20,34,35,36]. Hence, we sought to elucidate the function of *SfruGOX* specifically. Utilizing the CRISPR/Cas9 system, we produced *GOX* knock-out mutants following an established protocol [37]. The sgRNA was designed to target the second exon, the *SfruGOX*, and four different edited alleles were obtained through G1 generation (Figure 2A). After selective mating, a homozygous mutant (*SfruGO*^−/−^) was obtained for one of the alleles, which entailed a 2 bp deletion (Figure 2B). In congruence with the genetic manipulation, the GOX activity of salivary glands dissected from the mutant larvae was significantly lower than in the wild type, regardless of the diet of the larvae (Figure 2C). To investigate the impact of the *GOX* gene disruption on larvae growth, comparative analyses between WT and *SfruGOX*^−/−^ individuals were performed on B73 maize seedlings and artificial diets. As a result, no significant difference in growth rates between the WT and *SfruGOX*^−/−^ larvae was observed (Figure 2D). These results showed that *SfruGOX* was not required for *S. frugiperda* growth on maize under isolated laboratory conditions.

### 3.3. The Impact of SfruGOX on Maize Gene Expression

To elucidate the role of SfruGOX further, we conducted a comparative transcriptomics analysis on maize leaves attacked by either wild-type or *SfruGOX*^−/−^ mutant larvae, and maize leaves without insect infestation were included as the control group. Principal component analysis (PCA) results confirmed the high quality of the sequencing results (Figure S1). When the two larvae-infested groups were compared to the common un-infested control group, *SfruGOX*^−/−^ larvae elicited more DEGs (6566) than wild-type larvae (2610). Consistently, direct comparison of the two larvae-attacked groups identified 6248 DEGs, 3649 of which were up-regulated in the *SfruGOX*^−/−^ larvae-attacked leaves. These results suggest that the lack of *SfruGOX* attenuated the larvae’s ability to induce a weak plant transcriptional response. KEGG enrichment analysis of these 3649 DEGs revealed that defense-related secondary metabolic pathways, including alpha-linolenic acid metabolism, linoleic acid metabolism, terpenoid backbone biosynthesis, monoterpenoid biosynthesis, diterpenoid biosynthesis, and sesquiterpenoid and triterpenoid biosynthesis, were differentially regulated in maize leaves attacked by the two different *S. frugiperda* genotypes (Figures S4 and S5).

### 3.4. SfruGOX Suppresses Maize GLV Biosynthesis and Emission but Elicits Terpenoid Production

**Figure 3:**
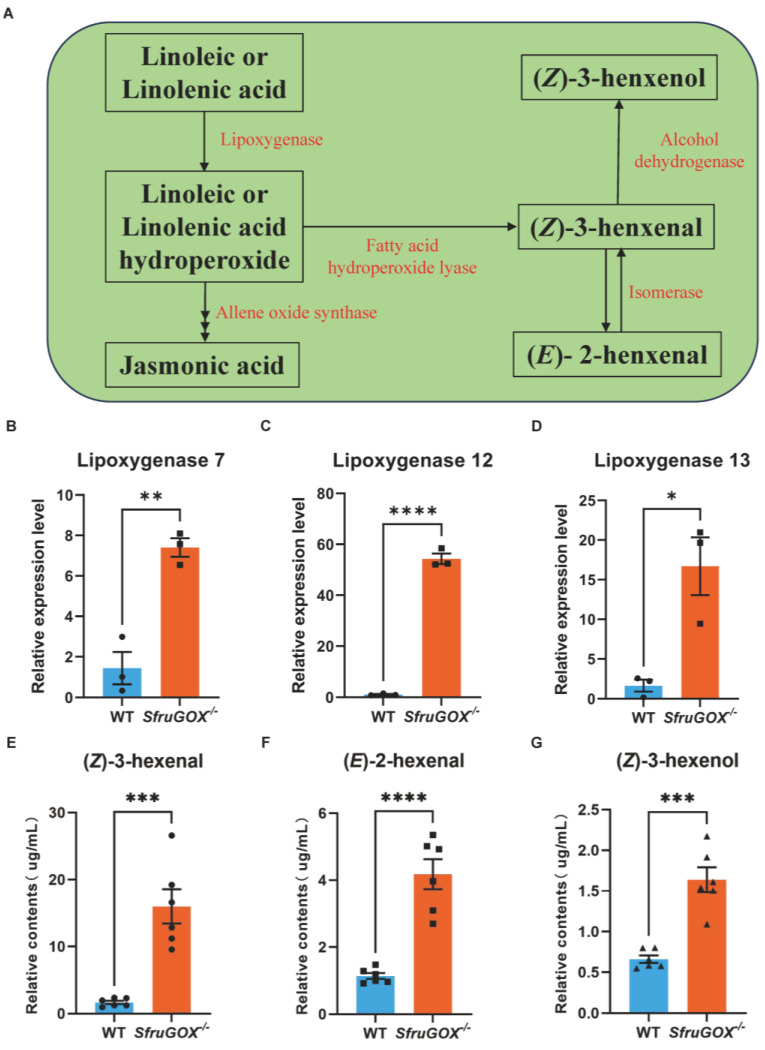
Comparison of GLVs induced by wild-type and mutant larvae feeding on maize and quantification of related synthetic genes at the transcriptional level. (**A**) The expression pattern of linolenic acid and linoleic acid pathway in wild-type and mutant larvae after feeding on maize. (**B**) Quantitative analysis of lipoxygenase 7 gene. (**C**) Quantitative analysis of lipoxygenase 12 gene. (**D**) Quantitative analysis of lipoxygenase 13 gene. (**E**) GC-MS detection of differences in (*Z*)-3-hexenal content induced by wild-type and mutant larvae. (**F**) GC-MS detection of differences in (*E*)-2-hexenal content induced by wild-type and mutant larvae. (**G**) GC-MS detection of differences in (*Z*)-3-hexenol content induced by wild-type and mutant larvae. The black squares, triangles and circles in the figure represent the number of repetitions. Mean ± SE is shown. Student’s *t*-test was used. *, *p* < 0.05, **, *p* < 0.01, ***, *p* < 0.001 ****, *p* < 0.0001.

Since the comparative transcriptomics analysis revealed linoleic acid and terpenoid biosynthetic pathways as the main differentially regulated pathways in maize attacked by *S. frugiperda* larvae with or without functional *SfruGOX*, these two pathways were further examined. The linoleic acid pathway is upstream to the production of GLVs (Figure 3A). The three DEGs identified in this pathway by RNA-seq were up-regulated in expression after feeding by *SfruGOX*^−/−^ larvae, and quantitative analysis of these gene by q-RT-PCR reconfirmed that the results showed a consistent pattern of changes in the expression of these genes (Figure 3B–D and Figure S6). Three GLVs, namely, (*E*)-2-hexenal, (*Z*)-3-hexenal, and (*Z*)-3-hexenol, were detected in higher concentrations in the maize seedling headspace when they were infested by the *SfruGOX*^−/−^ larvae (Figure 3E–G).

**Figure 4:**
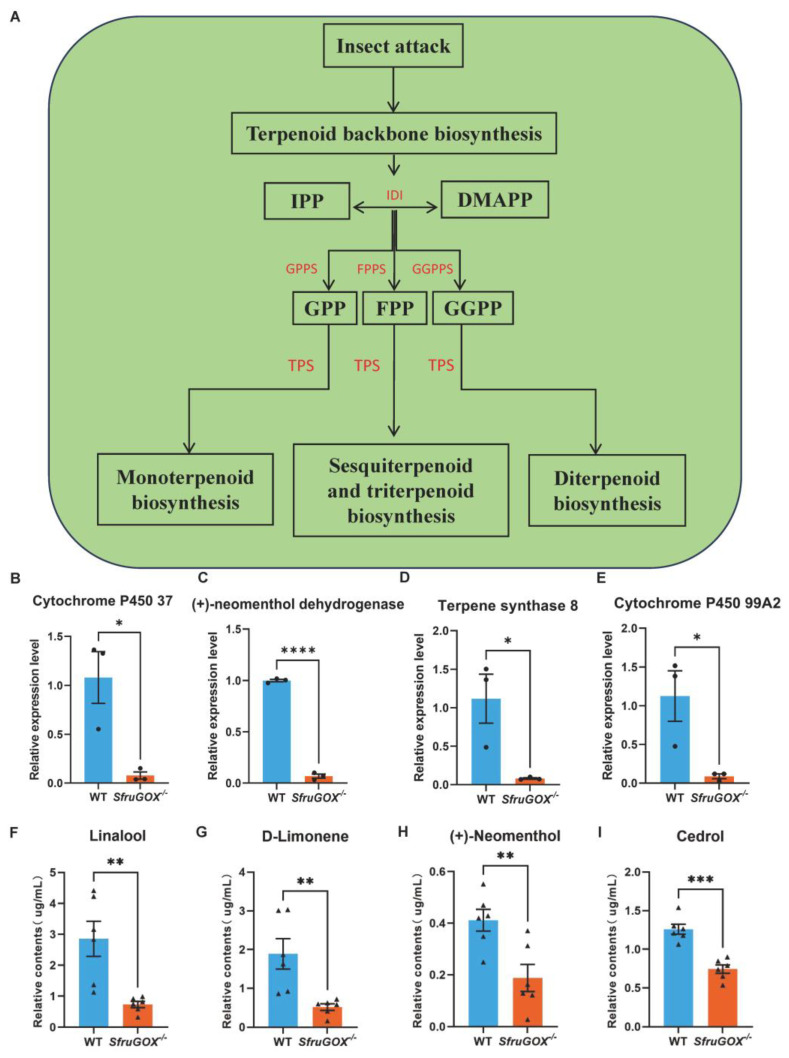
Comparison of terpenoid induction and quantitative analysis of related synthetic genes at the transcriptional level after feeding on maize in wild-type and mutant larvae. (**A**) Patterns of terpenoid synthesis pathways in wild-type and mutant larvae after feeding on maize. (**B**) Quantitative analysis of cytochrome P450 37 gene. (**C**) Quantitative fractionation of (+)-neomenthol dehydrogenase gene. (**D**) Quantitative analysis of terpene synthase 8 gene. (**E**) Cytochrome P450 99A2 gene quantification. (**F**) GC-MS detection of differences in linalool content induced by feeding on maize between wild-type and mutant larvae. (**G**) GC-MS detection of differences in D-limonene content induced by wild-type and mutant larvae feeding on maize. (**H**) GC-MS detection of differences in (+)-neomenthol content induced by wild-type and mutant larvae feeding on maize. (**I**) GC-MS detection of differences in cedrol content induced by wild-type and mutant larvae feeding on maize. Mean ± SE is shown. Student’s *t*-test was used. *, *p* < 0.05, **, *p* < 0.01, ***, *p* < 0.001 ****, *p* < 0.0001.

Terpenoid biosynthetic pathways were among the other significantly enriched metabolic pathways in maize leaves attacked by the two *S. frugiperda* genotypes (Figure S3 and Figure 4A). In contrast to the linoleic acid metabolic genes, terpene biosynthetic genes were more highly expressed in maize leaves attacked by wild-type larvae. This expression pattern was confirmed for four DEGs involved in terpene biosynthesis with q-RT-PCR (Figure 4B–E and Figure S6). In the same headspace GC-MS assay that detected the aforementioned GLVs, four terpene volatiles were identified. Intriguingly, the concentrations of these compounds were consistently higher for wild-type larvae-attacked seedlings, in accordance with the expression patterns of related terpene biosynthetic genes (Figure 4F–I).

## 4. Discussion

GOX is a common class of enzymes in insects, and has been reported in 91 species of insects from 24 families of Lepidoptera [38]. As a crucial component of oral secretion, GOX demonstrates varied expression in different tissues and developmental stages of insects, significantly influencing insect–plant interactions. The enzymatic activity of GOX, particularly evident in saliva, varies among Lepidoptera, with omnivorous insects generally exhibiting higher levels than specialists [38]. This variance may be attributable to differences in host plants or the insects’ own growth dynamics [19,39,40,41]. GOX in the OS of Lepidoptera (or expressed in salivary glands) has important functions for insect–plant interactions, and although the specific functions vary with insect–plant systems, the fact that there is a function is relatively conserved.

This investigation validated that the *SfruGOX* gene bears significant resemblance to its homologous counterparts in other insect species. Notably, SfruGOX has notably high expression levels in the salivary glands, especially during the fifth-instar larval stage. Previous research has indicated that GOX expression in *Helicoverpa armigera*, *Helicoverpa zea*, and *Spodoptera exigua* reaches its peak in fifth-instar larvae [39,40]. Furthermore, the *GOX* gene is expressed across various tissues in *H. armigera* and *H. zea*, including the foregut, midgut, fat body, and testis, with its highest expression level observed in the lower labial gland [41,42]. The expression pattern of the *SfruGOX* gene in *S. frugiperda*, as identified in this study, aligns closely with these findings.

In this research, the CRISPR/Cas9 system was employed to knock out the *GOX* gene in *S. frugiperda*. Subsequent analysis revealed significantly reduced GOX enzyme activity in the salivary glands of mutant larvae compared to wild-type larvae. These larvae were fed on maize and an artificial diet, yet no discernible differences in growth and feeding behavior were observed between wild-type and mutant larvae, similar to findings in *H. zea* [18].

Previous studies have found that the function of GOX is both positive and negative in different plant–insect interaction systems, that is, it is an elicitor and an effector. For example, the glucose oxidase GOX in the saliva of *H. zea* can inhibit the content of the defensive substance nicotine in the host plant tobacco while increasing the survival rate of *H. armigera* and promoting growth and development [34]. It can induce stomatal closure of tomato leaves and reduce the release of HIPVs [18]. It can also induce the response of the tomato proteinase inhibitor 2 (Pin2) defense gene, increase the density of trichomes, and cause the defense response of the JA pathway [20]. Here, we found that, even in the same system, the function of GOX is to inhibit GLV and activate terpenoids. The results of this study are consistent with the results reported by previous studies that caterpillar salivary GOX decreases green leaf volatile emission and increases terpene emission from maize, and in addition, *S. frugiperda* suppresses volatile emissions in maize [11,35]. This means that, depending on whether the natural enemy is affected by GLV or terpenoids, it is possible to induce different behavioral responses which may be beneficial or harmful to *S. frugiperda*. Therefore, our findings provide a potential molecular mechanism explanation for the “inconsistency” of GOX function in previous studies.

C6-alcohols have been consistently implicated in mediating indirect plant defenses within tritrophic interactions. Compelling evidence indicates that the vast majority of natural enemies are capable of detecting these volatile compounds and exhibiting positive responses [43]. The herbivore- or wound-induced compound (*Z*)-3-hexenol can exert direct effects on the physiology and behavior of phytophagous creatures through its inherent properties [43]. Additionally, linalool, synthesized by plants, provides a line of defense against insect pests and pathogens [44]. A seminal study demonstrated that the release of (*Z*)-3-hexenol and linalool, prompted by *Manduca sexta* larval activity, serves as an indirect defensive strategy by luring natural predatory enemies, thereby elevating mortality rates among herbivores in situ [45]. Subsequent research has highlighted (*Z*)-3-hexenol as not only a prevalent inducible chemical across ten plant species spanning seven families when afflicted by herbivory or mechanical damage, but also as a paramount infochemical for the attraction of parasitic wasps [46,47]. Moreover, earlier studies have confirmed the unique role of (*Z*)-3-hexenol in the host-seeking behavior of parasitoids [48].

In this study, we elucidated the GLVs emitted by maize during feeding by *S. frugiperda*, especially (*E*)-2-hexenal, (*Z*)-3-hexenal, and (*Z*)-3-hexenol, which are considered to have antibacterial properties and play a role in plant defense responses [49]. This process elicits a heightened defense reaction in maize against future attacks, underscoring the plant’s capacity to discern distress signals from damaged conspecifics and activate appropriate defense strategies to thwart infestation [50]. Moreover, *S. frugiperda* feeding prompts the emission of terpenoid compounds, including linalool, in response to insect-induced damage [51]. Our findings indicate that wild-type *S. frugiperda* larvae induce an increase in the production of specific terpenoid volatiles such as linalool, D-limonene, (+)-neomenthol, and cedrol, concurrently suppressing the generation of GLVs like (*E*)-2-hexenal, (*Z*)-3-hexenal, and (*Z*)-3-hexenol.

## 5. Conclusions

By generating a genetic knockout *S. frugiperda* line lacking a functional *SfruGOX*, we demonstrated that this gene is not required for larvae survival on maize per se, but has a profound influence on the HIPV profile of maize upon *S. frugiperda* larvae attack. These alterations in HIPVs could influence maize’s indirect defense and impact the performance of larvae under field conditions, where proper natural enemies of *S. frugiperda* are present.

## Data Availability

Transcriptome data are deposited in the SRA (BioProject PRJNA1105851). Other data are contained within the article and Supplementary Materials.

## Supplementary Materials

The following supporting information can be downloaded at: https://www.mdpi.com/article/10.3390/insects15070511/s1, Figure S1: principal component analysis of mutant, wild type and control; Figure S2: volcano plot of differential genes comparing wild type and mutant for maize infestation; Figure S3: histogram of differential genes in mutant, wild type and control; Figure S4: Differential gene KEGG enrichment in wild type vs. mutant (mutant up-regulated expression); Figure S5: Differential gene KEGG enrichment in wild type vs. mutant (mutant down-regulated expression); Figure S6. FPKM value of differential genes in maize leaves after feeding by wild-type and mutant larvae of *S. frugiperda*; Table S1: The GenBank accession numbers are listed; Table S2: The list of qRT-PCR primer.
