# Supplementary material for: Spodoptera frugiperda Salivary Glucose Oxidase Reduces the Release of Green Leaf Volatiles and Increases Terpene Emission from Maize
Source: Insects. 2024 Jul 8;15(7):511. doi: 10.3390/insects15070511 (PMC11277060; doi:10.3390/insects15070511)
Supplement: Supplementary file 1 [file insects-15-00511-s001.zip › insects-3014014-supplementary.pdf]

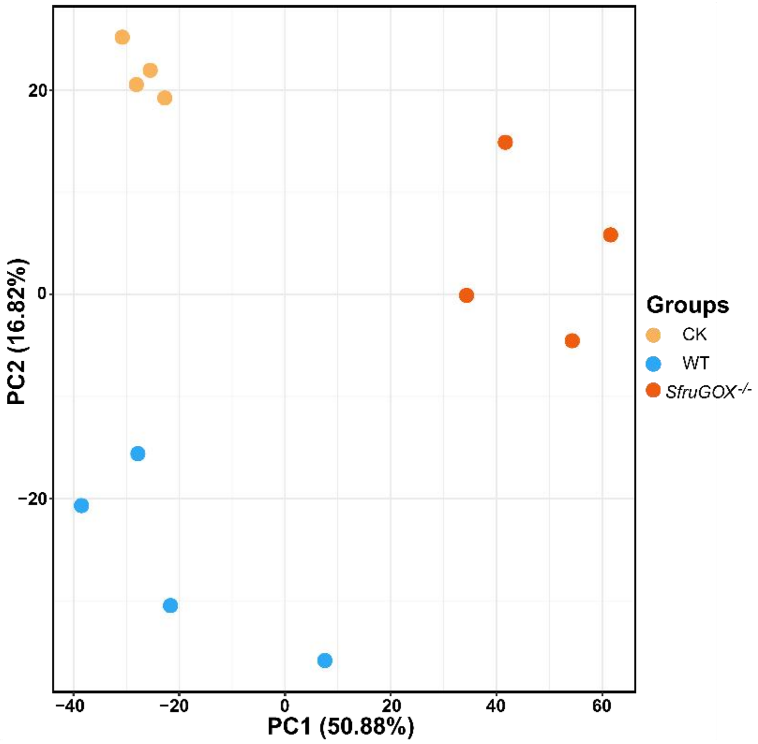

Figure S1. principal component analysis of mutant, wild type and control. In the graph, the horizontal axis represents the first principal component, and the vertical axis represents the second principal component; yellow denotes corn that has not been consumed (CK), blue indicates corn consumed by wild-type larvae (WT), and red signifies corn consumed by mutant larvae (*SfruGOX*$^{-/-}$).

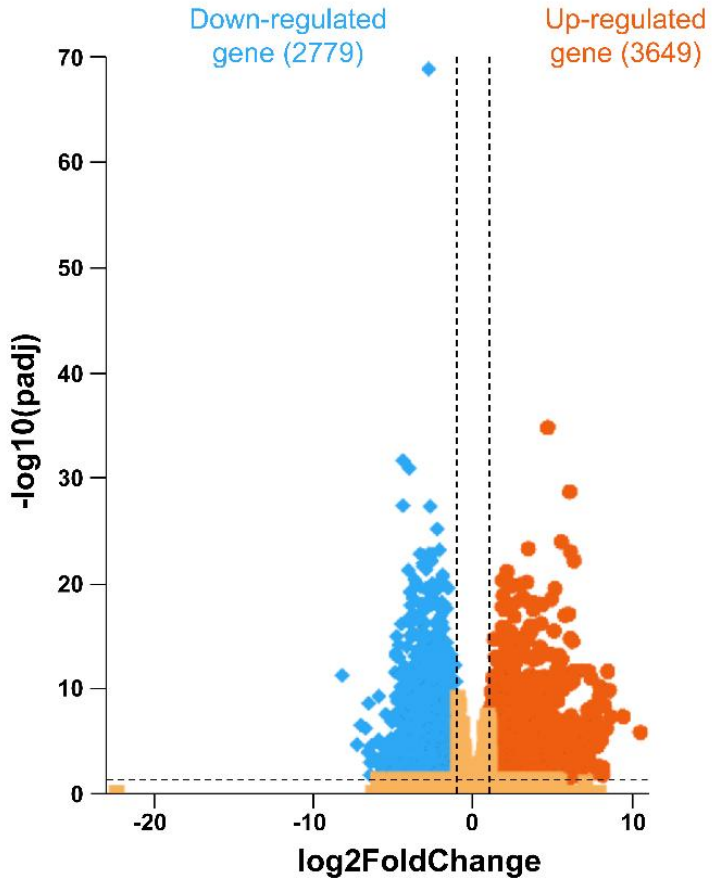

Figure S2. volcano plot of differential genes comparing wild type and mutant for maize infestation. The horizontal axis represents the logarithmic fold change in gene expression (log2FoldChange) between corn groups subjected to feeding treatments by mutant larvae and those by wild-type larvae. The vertical axis indicates the level of significance in the differential expression between the two groups (-log10padj). The dashed line delineates the threshold criteria for differential gene selection (|log2FoldChange| > 1 and -log10padj < 0.05). Red dots signify genes significantly upregulated in the mutant, blue dots represent genes significantly downregulated in the mutant, and yellow dots denote genes with no significant change in expression.

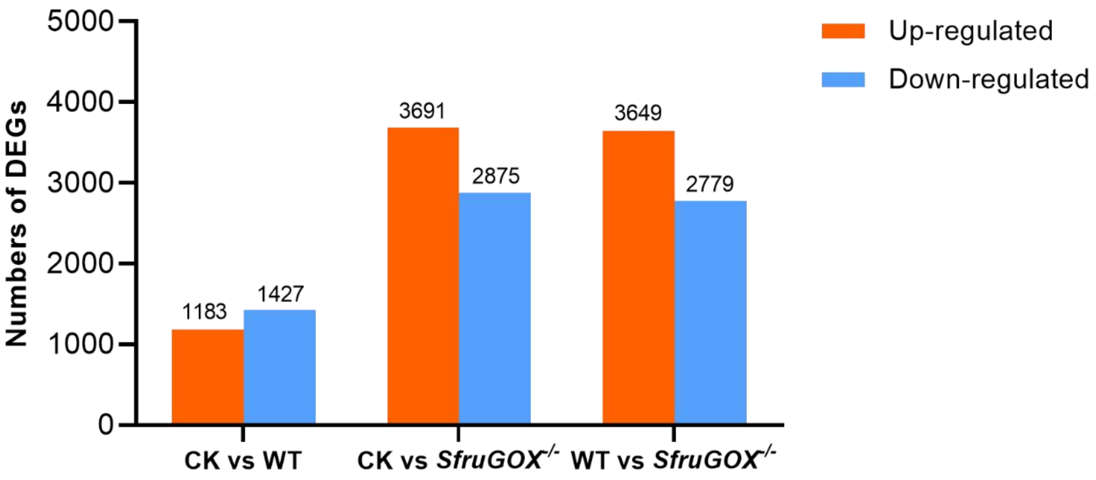

Figure S3. histogram of differential genes in *SfruGOX*$^{-/-}$ mutant, wild type (WT) and control(CK). Red and blue represent upregulated and downregulated differential genes, respectively, with the numbers atop the bars indicating the count of differential genes.

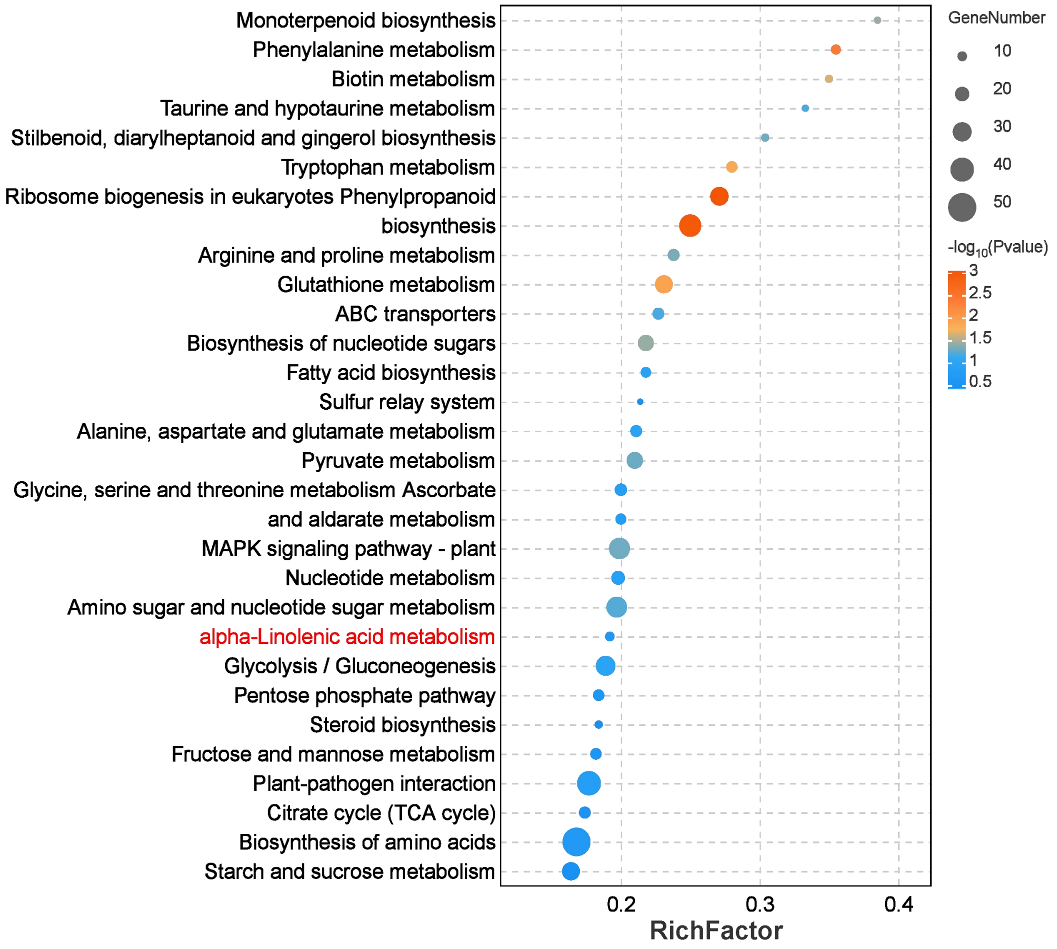

Figure S4. Differential gene KEGG enrichment in wild type vs. mutant (mutant up-regulated expression). The horizontal coordinate is the ratio of the number of differential genes annotated to the KEGG pathway to the total number of differential genes, and the vertical coordinate is the KEGG pathway; The size of the dots indicates the number of genes enriched in the KEGG Term, with blue representing smaller -log10pvalue and red indicating larger -log10pvalue. Pathways related to the synthesis of GLVs are marked in red

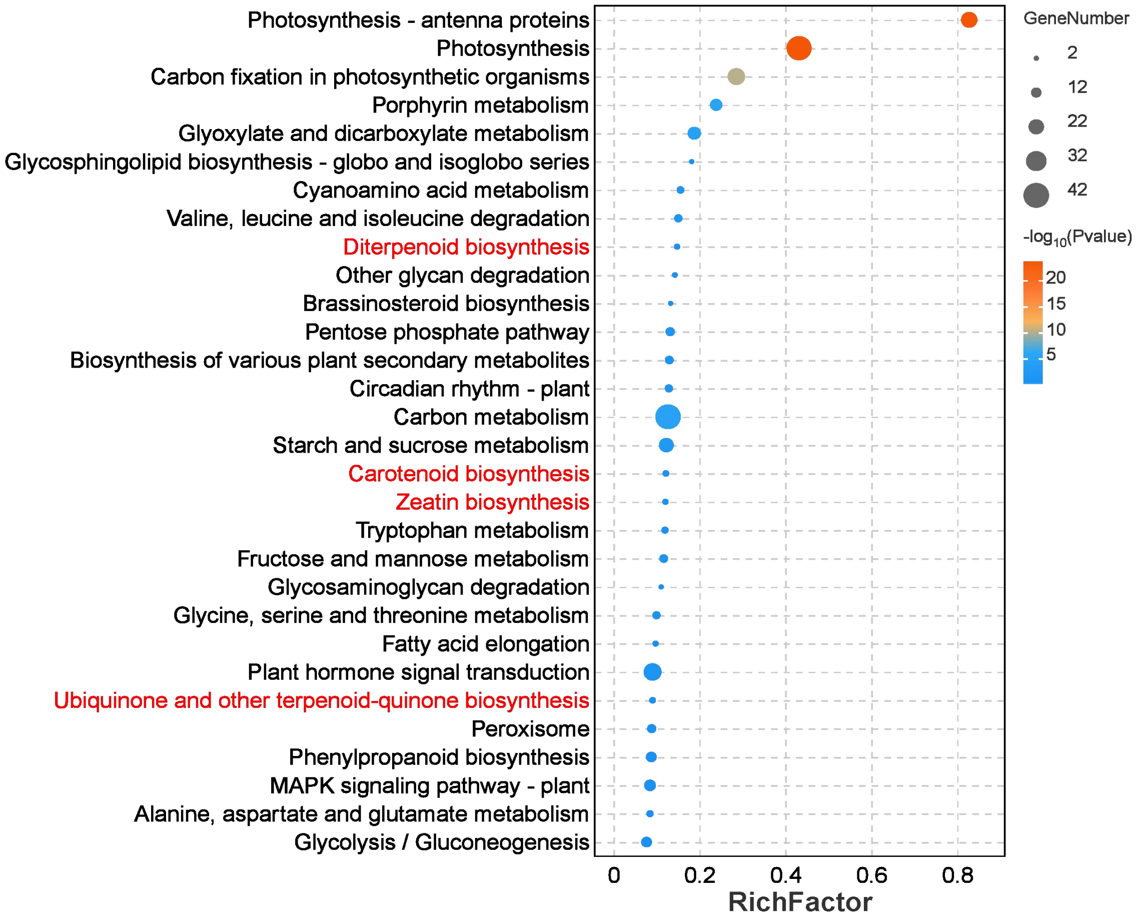

Figure S5. Differential gene KEGG enrichment in wild type vs. mutant (mutant down-regulated expression). The horizontal coordinate is the ratio of the number of differential genes annotated to the KEGG pathway to the total number of differential genes, and the vertical coordinate is the KEGG pathway; The size of the dots represents the number of genes enriched in the KEGG Term, with blue indicating smaller -log10pvalue and red representing larger -log10pvalue. Pathways related to the synthesis of terpenoid compounds are marked in red.

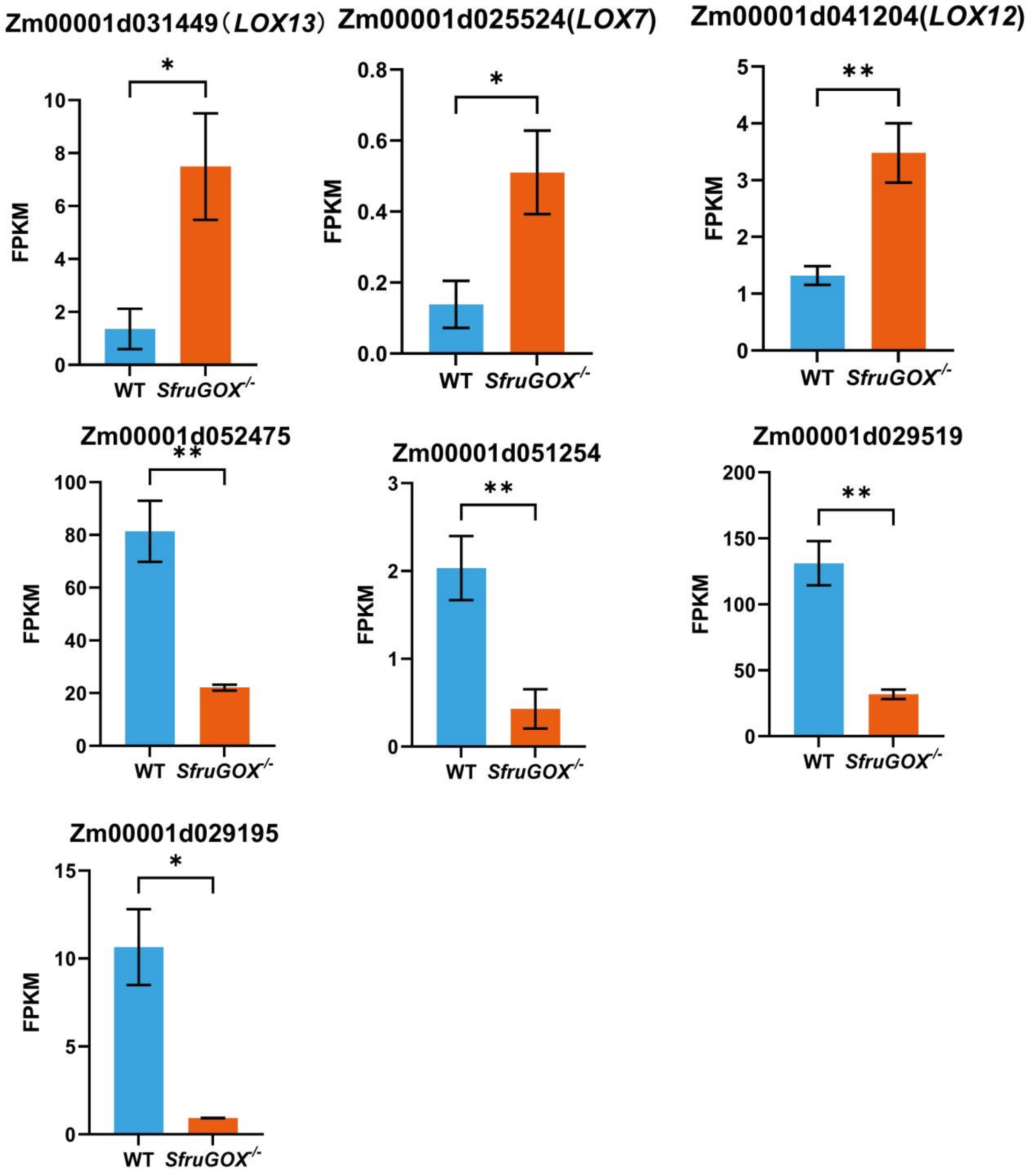

Figure S6. FPKM value of differential genes in maize leaves after feeding by wild-type and mutant larvae of *S.frugiperda*. Student's t-test was used. *, $p < 0.05$, **, $p < 0.01$.

Table S1. The GenBank accession numbers are listed

Species name	Abbreviation of species names	Accession
Spodoptera frugiperda	SfruGOX	XP_035448057.2
Spodoptera litura	SlitGOX	XP_022831394.1
Spodoptera exigua	SexiGOX	ADL38963.1
Heliothis viriplaca	HvirGOX	ACJ71598.1
Helicoverpa zea	HzeaGOX	ACJ71598.1
Helicoverpa armigera	HarmGOX	ACC94296.1
Mythimna separata	MsepGOX	ASF79657.1
Bombyx mori	BmorGOX	XP_012548096.1
Ostrinia furnacalis	OfurGOX	XP_028157691.1
Chilo suppressalis	CsupGOX:	RVE46880.1
Drosophila melanogaster	DmelGOX	NP_001350861.1

Table S2. The list of qRT-PCR primer

Primer name	Sequence5'-3'
RPL10-F	TGGGTAAGAAGAAGGCTACG
RPL10-R	TGTTGATGCGGATGACAT
a-TUB-F	AGGGCTGTGTTTGTTGACT
a-TUB-R	TCCTTACCGATGGTGTAGTG
SOD-F	TCGGCACAATCATCAGTC
SOD-R	AGTCCTTCTCAATAGCCTGC
GOX.1-F:	AACCGATCCCTTCGAGCAAG
GOX.1-R:	GGCGCCACTTTATCGCATTT
GOX.2-F:	CCTGAGGATTGTGGGTGTCC
GOX.2-R:	TGCATGGGGTCGATATGCTG
EF1α-F	TGGGCCTACTGGTCTTACTACTGA
EF1α-R	ACATACCCACGCTTCAGATCCT
A-TUB-F	CTACCTCACGGCATCTGCTATGT
A-TUB-R	GTCACACACACTCGACTTCACG
Zm00001d031449-F	CCGTGGAGCCCTACATCATC
Zm00001d031449-R	GCTCTATGGCGTACCTCTGC
Zm00001d041204-F	ACCATCAGCTCATCAGCCAC
Zm00001d041204-R	CATGGTCCTCTCCAGGATGC
Zm00001d025524-F	GAGTACCCGGAGCCCATCTA
Zm00001d025524-R	GGCCCTCCTTGAAGAGGTTG
Zm00001d051254-F	GAAAGAGGAGCTCAGCAGCA
Zm00001d051254-R	CTTCGCAAGGATCCGGGAAT
Zm00001d029519-F	CGCTACACCAAGACGGTGAT
Zm00001d029519-R	TTCAGGCATGAACCTGTCGG
Zm00001d029195-F	CTGGAATCGATACGACGGGG
Zm00001d029195-R	GCGAGCTCCAGTATCTCAGG
Zm00001d052475-F	CTGTGGCGCCAGTAAGAAGA
Zm00001d052475-R	GGTGTCTCGGTGAGGAACTT
